# Supplementary material for: Tuberculosis-Associated Hemophagocytic Lymphohistiocytosis: A Review of Current Literature
Source: J Clin Med. 2023 Aug 18;12(16):5366. doi: 10.3390/jcm12165366 (PMC10455670; doi:10.3390/jcm12165366)
Supplement: Supplementary file 1 [file jcm-12-05366-s001.zip › jcm-2526568-supplementary.pdf]

Journal of
Clinical Medicine

MDPI

Supplementary file for paper:

Tuberculosis-Associated Hemophagocytic Lymphohistiocytosis: A Review of Current Literature

Trym Fauchald [1], Bjørn Blomberg [2,3] and Håkon Reikvam [2,3,*]

[1] Faculty of Medicine, University of Bergen, 5007 Bergen, Norway; trym.fauchald@student.uib.no
[2] Department of Clinical Science, University of Bergen, 5007 Bergen, Norway; bjorn.blomberg@uib.no
[3] Department of Medicine, Haukeland University Hospital, 5021 Bergen, Norway
* Correspondence: hakon.reikvam@uib.no; Tel.: +47-55-97-29-97

Supplementary Table S1. HLH-2004 criteria *

**The diagnosis HLH can be established if one of either 1 or 2 below is fulfilled:**
1: a molecular diagnosis consistent with HLH
2: diagnostic criteria of HLH fulfilled (at least 5 of 8)
1. Fever
2. Splenomegaly
3. Cytopenias (affecting ≥2 of 3 lineages in peripheral blood)
Hemoglobin <9 g/dL
Platelets <100 $\times 10^9/L$
Neutrophils <1.0 $\times 10^9/L$
4. Hypertriglyceridemia and/or hypofibrinogenemia
Fasting triglycerides ≥3.0 mmol/L
Fibrinogen ≤1.5 g/L
5. Hemophagocytosis in bone marrow, spleen, lymph nodes, or liver
(With no sign of malignancy, in the case of familial HLH)
6. Low or absent NK-cell activity
7. Ferritin >500 μg/L †
8. Elevated s-CD25 (α-chain of IL-2 receptor) ‡

* Adapted from Henter et al. [1] and Jordan et al. [2].
† Most patients with HLH display significantly higher levels of ferritin than 500 μg/L; levels above 2-3000 μg/L are considered concerning of HLH, and above 10 000 μg/L is highly suspicious
‡ Elevations above age adjusted, laboratory-specific normal levels (defined as >2 standard deviations (SD) from the mean) appear more meaningful than the original designation of >2400 U/L because of variations between laboratories.

Supplementary Table S2. HScore for reactive hemophagocytic syndrome [1]

Parameter	Criteria for score	No. of points
Known underlying immuno-	No	0
suppression [2]	Yes	+18
Temperature, °C	<38.4	0
	38.4-39.4	+33
	>39.4	+49
Organomegaly	No	0

	Hepato- or splenomegaly	+23
	Hepatosplenomegaly	+38
Number of cytopenias [3]	1 lineage	0
	2 lineages	+24
	3 lineages	+34
Ferritin, μg/L	<2000	0
	2000-6000	+35
	>6000	+50
Triglyceride, mg/dL (mmol/L)	<132.7 (<1.5)	0
	132.7-354 (1.5-4.0)	+44
	>354 (>4.0)	+64
Fibrinogen, mg/dL (g/L)	>250 (>2.5)	0
	≤250 (≤2.5)	+30
Aspartate aminotransferase (AST), U/L	<30	0
	≥30	+19
Hemophagocytosis features on bone marrow aspirate	No	0
	Yes	+35

[1] Adapted from Fardet et al. [3].
[2] HIV positive or receiving long-term immunosuppressive therapy (i.e. glucocorticoids, cyclosporine, azathioprine)
[3] Cytopenias defined as hemoglobin ≤ 9.2 g/dL, leukocyte count $\leq 5 \times 10^9/L$, and platelets $\leq 110 \times 10^9/L$

Supplementary Table S3 Summary of diagnostic criteria per patient.

Patient	Sex	Age	Comorbidity	Fever	Cytopenia	Splenomegaly	Ferritin	Triglycerides/fibrinogen	Reduced NK-cell activity	Elevated s-IL2r	Hemophagocytosis	Sum HLH-04 criteria	HScore	Treatment	Outcome	Reference
1	M	28	Schistosomiasis	Yes	Yes	Yes	Yes	Yes	N/A	N/A	Yes	6	171	Both	Death	[4]
2	M	54	HT	Yes	Yes	Yes	Yes	Yes	No	Yes	Yes	7	254	Both	Recovery	[5]
3	M	44	AS	Yes	Yes	N/A	Yes	N/A	N/A	N/A	Yes	4	179	Both	Recovery	[6]
4	F	35		Yes	Yes	Yes	N/A	Yes	N/A	N/A	Yes	5	223	Both	Recovery	[7]
5	M	61	HT, DM, CAD	Yes	Yes	N/A	Yes	Yes	N/A	N/A	Yes	5	235	Both	Recovery	[8]
6	M	46	HIV/AIDS, EBV	Yes	Yes	N/A	N/A	N/A	N/A	N/A	Yes	3	120	Both	Death	[9]
7	F	60		Yes	Yes	Yes	Yes	Yes	Yes	N/A	Yes	7	252	Both	Death	[10]
8	F	31		Yes	Yes	N/A	Yes	Yes	Yes	N/A	Yes	6	281	Both	Death	[10]
9	F	78		Yes	Yes	Yes	Yes	Yes	Yes	N/A	Yes	7	259	immune	Death	[10]
10	M	32		Yes	Yes	Yes	Yes	Yes	N/A	Yes	Yes	7	244	Both	Recovery	[10]
11	M	37		Yes	Yes	Yes	Yes	Yes	Yes	N/A	Yes	7	284	Both	Death	[10]
12	F	28		Yes	Yes	N/A	Yes	Yes	Yes	Yes	Yes	7	271	Both	Recovery	[10]

Patient	Sex	Age	Comorbidity	Fever	Cytopenia	Splenomegaly	Ferritin	Triglycerides/fibrinogen	Reduced NK-cell activity	Elevated s-IL2r	Hemophagocytosis	Sum HLH-04 criteria	HScore	Treatment	Outcome	Reference
13	F	23		Yes	Yes	Yes	Yes	Yes	N/A	N/A	Yes	6	269	ATT	Death	[10]
14	F	60		Yes	Yes	Yes	Yes	Yes	Yes	Yes	Yes	8	269	Both	Death	[10]
15	F	19		Yes	Yes	Yes	Yes	Yes	N/A	Yes	Yes	7	219	Both	Recovery	[11]
16	M	<1	SD	Yes	Yes	Yes	Yes	N/A	N/A	N/A	Yes	5	174	Both	Death	[12]
17	M	<1		Yes	No	Yes	Yes	Yes	N/A	N/A	Yes	5	204	Both	Recovery	[13]
18	F	14		Yes	Yes	No	Yes	Yes	No	N/A	Yes	5	201	Both	Recovery	[14]
19	F	<1		Yes	Yes	Yes	N/A	N/A	N/A	N/A	Yes	4	159	Both	Recovery	[15]
20	M	15		Yes	Yes	No	N/A	N/A	N/A	N/A	No	2	109	Both	Recovery	[16]
21	M	17		Yes	Yes	Yes	Yes	Yes	N/A	N/A	Yes	6	184	Both	Recovery	[17]
22	F	9		Yes	Yes	Yes	N/A	N/A	N/A	N/A	Yes	4	175	Both	Recovery	[18]
23	F	<1		Yes	Yes	Yes	Yes	N/A	N/A	N/A	Yes	5	228	None	Death	[19]
24	M	41	GPA	Yes	Yes	Yes	Yes	N/A	N/A	N/A	Yes	5	212	Both	Recovery	[20]
25	M	20		Yes	No	Yes	No	Yes	N/A	N/A	Yes	4	208	Both	Recovery	[21]
26	M	57		Yes	No	Yes	No	Yes	N/A	N/A	Yes	4	224	ATT	Death	[21]
27	M	11		Yes	Yes	N/A	Yes	No	N/A	N/A	Yes	4	235	Both	Recovery	[22]
28	M	22	*C. neoformans*	Yes	Yes	Yes	Yes	N/A	Yes	Yes	N/A	6	174	ATT	Recovery	[23]
29	M	23		Yes	Yes	Yes	Yes	N/A	N/A	N/A	Yes	5	238	Both	Recovery	[24]
30	M	63	RF, HT, CAD, DM, EBV	Yes	Yes	Yes	Yes	Yes	N/A	Yes	Yes	7	274	Both	Death	[25]
31	M	58	RF, DM	Yes	Yes	Yes	Yes	Yes	N/A	N/A	Yes	6	243	Both	Death	[26]
32	F	74		N/A	No	Yes	Yes	Yes	N/A	Yes	Yes	5	103	Both	Recovery	[27]
33	F	58	HT, lymphoma	Yes	Yes	Yes	Yes	Yes	N/A	N/A	Yes	6	240	immune	Death	[28]
34	M	<1		Yes	Yes	Yes	Yes	Yes	N/A	N/A	Yes	6	273	Both	Death	[29]
35	M	<1		Yes	Yes	Yes	Yes	Yes	No	Yes	Yes	7	200	Both	Recovery	[30]
36	M	50	RF, DM	Yes	Yes	Yes	Yes	Yes	N/A	N/A	Yes	6	288	ATT	Death	[31]
37	M	36	RF	Yes	Yes	Yes	Yes	N/A	N/A	Yes	Yes	6	203	None	Death	[31]
38	M	56	RF, DM	Yes	Yes	Yes	Yes	N/A	N/A	N/A	Yes	5	238	None	Death	[31]
39	F	<1		Yes	Yes	Yes	Yes	Yes	N/A	N/A	Yes	6	214	Both	Recovery	[32]
40	F	27	IBD, PCOS, ADS	Yes	N/A	Yes	Yes	Yes	N/A	N/A	Yes	5	153	Both	Recovery	[33]
41	F	54	EPC	Yes	Yes	N/A	Yes	N/A	Yes	N/A	Yes	5	182	Both	Recovery	[34]
42	F	80	MR, cervical prolapse	Yes	Yes	Yes	Yes	Yes	N/A	Yes	Yes	7	229	Both	Recovery	[35]
43	M	17		Yes	Yes	Yes	Yes	No	N/A	N/A	No	4	130	ATT	Recovery	[36]
44	M	28		Yes	Yes	Yes	Yes	Yes	N/A	Yes	Yes	7	321	Both	Death	[37]
45	F	40		Yes	Yes	Yes	Yes	Yes	N/A	N/A	Yes	6	303	Both	Recovery	[38]
46	M	22	ESRD	Yes	Yes	N/A	Yes	N/A	N/A	N/A	Yes	4	152	Both	Recovery	[39]

Patient	Sex	Age	Comorbidity	Fever	Cytopenia	Splenomegaly	Ferritin	Triglycerides/fibrinogen	Reduced NK-cell activity	Elevated s-IL2r	Hemophagocytosis	Sum HLH-04 criteria	HScore	Treatment	Outcome	Reference
47	F	36	SLE	Yes	Yes	N/A	N/A	N/A	N/A	N/A	Yes	3	136	Both	Death	[40]
48	F	68	DM	Yes	No	Yes	N/A	Yes	N/A	N/A	Yes	4	140	None	Death	[41]
49	M	59	DM	Yes	Yes	Yes	N/A	N/A	N/A	N/A	Yes	4	130	None	Death	[41]
50	M	76	SCLC	Yes	Yes	Yes	N/A	N/A	N/A	N/A	Yes	4	130	None	Death	[41]
51	M	75	RF	Yes	Yes	N/A	Yes	Yes	N/A	N/A	Yes	5	200	Both	Death	[42]
52	M	50	AI	Yes	Yes	Yes	Yes	N/A	N/A	N/A	Yes	5	194	Both	Death	[43]
53	M	36		Yes	Yes	No	Yes	N/A	Yes	Yes	Yes	6	201	Both	Recovery	[44]
54	M	25		Yes	Yes	Yes	Yes	Yes	Yes	N/A	No	6	258	Both	Recovery	[45]
55	F	26	Pregnant, ES	Yes	Yes	Yes	Yes	Yes	Yes	Yes	Yes	8	273	Both	Recovery	[46]
56	M	14		Yes	Yes	Yes	Yes	Yes	Yes	N/A	No	6	204	Both	Recovery	[47]
57	M	70		Yes	Yes	Yes	No	Yes	N/A	N/A	Yes	5	170	None	Death	[48]
58	M	43	HIV/AIDS	Yes	Yes	N/A	Yes	N/A	N/A	N/A	Yes	4	160	ATT	Death	[49]
59	M	22		Yes	Yes	N/A	N/A	N/A	N/A	N/A	Yes	3	102	Both	Recovery	[50]
60	M	40		Yes	Yes	Yes	N/A	N/A	N/A	N/A	Yes	4	140	ATT	Recovery	[51]
61	M	43		Yes	Yes	Yes	N/A	Yes	N/A	N/A	Yes	5	161	Both	Recovery	[52]
62	M	34		Yes	Yes	Yes	Yes	Yes	N/A	N/A	Yes	6	288	Both	Death	[53]
63	M	52		Yes	Yes	No	N/A	N/A	N/A	N/A	Yes	3	108	Both	Recovery	[54]
64	F	48	T-PLL	Yes	Yes	Yes	Yes	N/A	N/A	N/A	Yes	5	209	immune	Death	[55]
65	F	73	RF	Yes	Yes	Yes	N/A	N/A	N/A	N/A	Yes	4	159	ATT	Recovery	[56]
66	M	38		Yes	Yes	Yes	N/A	N/A	N/A	N/A	Yes	4	140	Both	Recovery	[57]
67	F	18		Yes	Yes	Yes	Yes	Yes	N/A	N/A	Yes	6	268	Both	Recovery	[58]
68	M	68		Yes	Yes	Yes	N/A	Yes	N/A	N/A	Yes	5	205	Both	Recovery	[59]
69	F	67		Yes	No	N/A	N/A	Yes	N/A	N/A	Yes	3	117	Both	Death	[59]
70	M	39		Yes	Yes	No	Yes	N/A	N/A	Yes	Yes	5	146	Both	Recovery	[60]
71	M	42		Yes	Yes	Yes	Yes	Yes	N/A	No	Yes	6	254	Both	Recovery	[61]
72	M	<1		Yes	Yes	Yes	Yes	Yes	Yes	N/A	No	6	189	Both	Recovery	[62]
73	F	83	RF, HT, CVA	Yes	Yes	Yes	N/A	Yes	N/A	N/A	Yes	5	208	None	Death	[63]
74	M	63	DM	Yes	Yes	Yes	Yes	Yes	N/A	Yes	Yes	7	258	Both	Death	[64]
75	F	35	ALL	Yes	Yes	Yes	Yes	Yes	N/A	N/A	Yes	6	202	Both	Recovery	[65]
76	F	4	IFN-γ-r dysfunction	Yes	Yes	Yes	Yes	Yes	N/A	N/A	No	5	219	Both	Death	[66]
77	F	2		Yes	Yes	Yes	Yes	Yes	N/A	N/A	Yes	6	223	Both	Recovery	[67]
78	M	49		Yes	Yes	N/A	Yes	Yes	N/A	Yes	Yes	6	223	Both	Death	[68]
79	M	27		Yes	Yes	Yes	Yes	Yes	N/A	N/A	Yes	6	258	ATT	Death	[69]
80	M	<1		Yes	Yes	Yes	Yes	Yes	N/A	Yes	Yes	7	268	Both	Recovery	[70]
81	M	63	HCV, LTtx	Yes	Yes	No	Yes	Yes	N/A	N/A	Yes	5	253	Both	Death	[71]

Patient	Sex	Age	Comorbidity	Fever	Cytopenia	Splenomegaly	Ferritin	Triglycerides/fibrinogen	Reduced NK-cell activity	Elevated s-IL2r	Hemophagocytosis	Sum HLH-04 criteria	HScore	Treatment	Outcome	Reference
82	F	31	Lymphoma	N/A	Yes	Yes	N/A	N/A	N/A	N/A	Yes	3	110	Both	Death	[72]
83	M	28	Mb. Crohn	Yes	Yes	N/A	Yes	Yes	N/A	N/A	Yes	5	150	Both	Recovery	[73]
84	F	56	RF, MDS	Yes	Yes	Yes	Yes	N/A	N/A	Yes	Yes	6	225	Both	Recovery	[74]
85	F	53	HIV/AIDS	Yes	Yes	No	N/A	N/A	N/A	N/A	Yes	3	143	Both	Death	[75]
86	F	71	Seronegative arthritis	Yes	Yes	Yes	Yes	Yes	N/A	N/A	Yes	6	291	Both	Death	[76]
87	M	56		Yes	Yes	N/A	Yes	No	N/A	Yes	Yes	5	152	Both	Recovery	[77]
88	M	67	Aortoiliac bypass	Yes	Yes	No	N/A	N/A	N/A	N/A	Yes	3	92	None	Death	[78]
89	M	37	KTtx, RF	Yes	Yes	No	N/A	N/A	N/A	N/A	Yes	3	143	None	Death	[79]
90	M	83		Yes	No	No	No	No	N/A	N/A	Yes	3	112	ATT	Recovery	[80]
91	M	70	CML	Yes	Yes	No	Yes	No	N/A	N/A	Yes	4	118	ATT	Recovery	[80]
92	M	29		Yes	Yes	Yes	Yes	Yes	N/A	N/A	No	5	238	Both	Recovery	[81]
93	F	29		Yes	No	No	N/A	N/A	N/A	N/A	Yes	2	115	ATT	Recovery	[82]
94	M	8	Malaria	Yes	Yes	Yes	Yes	Yes	N/A	N/A	Yes	6	244	ATT	Recovery	[83]
95	F	20	Pregnant	Yes	Yes	Yes	Yes	Yes	N/A	N/A	Yes	6	224	Both	Recovery	[84]
96	M	40	Candida	Yes	Yes	Yes	Yes	N/A	N/A	N/A	Yes	5	209	Both	Recovery	[85]
97	M	46		Yes	No	Yes	Yes	N/A	N/A	N/A	Yes	4	149	ATT	Recovery	[85]
98	M	42	Sarcoidosis	Yes	Yes	Yes	N/A	N/A	N/A	N/A	Yes	4	125	Both	Death	[86]
99	F	69	NPC	Yes	Yes	N/A	N/A	N/A	N/A	N/A	Yes	3	111	None	Death	[87]
100	F	78	HT, hip fx	Yes	Yes	N/A	Yes	Yes	N/A	N/A	Yes	5	245	ATT	Recovery	[88]
101	M	80	AF, smoker	Yes	Yes	N/A	Yes	Yes	N/A	N/A	Yes	5	132	ATT	Death	[88]
102	M	17		Yes	Yes	Yes	Yes	No	N/A	N/A	Yes	5	200	ATT	Recovery	[89]
103	M	40	Alcoholism	Yes	Yes	Yes	Yes	Yes	N/A	N/A	N/A	5	204	Both	Recovery	[90]
104	M	59	Lymphoma, smoker, alcoholism	Yes	Yes	Yes	N/A	N/A	N/A	N/A	Yes	4	175	ATT	Death	[91]
105	M	29		Yes	Yes	Yes	Yes	Yes	N/A	N/A	N/A	5	159	ATT	Death	[92]
106	F	47	SLE	Yes	Yes	Yes	Yes	N/A	N/A	N/A	Yes	5	217	Both	Death	[93]
107	M	22		Yes	Yes	No	N/A	N/A	N/A	N/A	Yes	3	144	ATT	Recovery	[94]
108	F	60	HT, DM	Yes	Yes	No	N/A	N/A	N/A	N/A	Yes	3	102	ATT	Death	[95]
109	F	14		Yes	No	Yes	N/A	Yes	N/A	N/A	Yes	4	196	Both	Recovery	[96]
110	X	45	Lymphoma, KTtx	Yes	Yes	No	Yes	Yes	N/A	N/A	Yes	5	223	N/A	Recovery	[97]
111	X	44	KTtx	Yes	Yes	Yes	N/A	Yes	N/A	N/A	Yes	5	231	N/A	Death	[97]
112	M	42	KTtx	Yes	Yes	No	N/A	Yes	N/A	N/A	Yes	4	150	ATT	Death	[98]
113	F	X		Yes	Yes	Yes	Yes	No	N/A	N/A	Yes	5	160	ATT	Death	[99]
114	F	75		Yes	Yes	N/A	Yes	Yes	N/A	N/A	Yes	5	167	N/A	Death	[100]
115	F	60		Yes	N/A	No	N/A	N/A	N/A	N/A	Yes	2	110	ATT	Recovery	[101]

Patient	Sex	Age	Comorbidity	Fever	Cytopenia	Splenomegaly	Ferritin	Triglycerides/fibrinogen	Reduced NK-cell activity	Elevated s-IL2r	Hemophagocytosis	Sum HLH-04 criteria	HScore	Treatment	Outcome	Reference
116	F	<1		Yes	N/A	Yes	N/A	N/A	N/A	N/A	Yes	3	125	ATT	Recovery	[102]

X, age or gender unknown/unspecified.

N/A, data not available.

AS, ankylosing spondylitis; HT, hypertension; DM, diabetes mellitus; CAD, coronary artery disease; EBV, Epstein-Barr virus; SD, seborrheic dermatitis; GPA, granulomatosis with polyangiitis; C. neoformans, Cryptococcus neoformans; RF, renal failure; IBD, inflammatory bowel disease; PCOS, polycystic ovary syndrome; ADS, anxiety-depressive syndrome; EPC, epilepsia partialis continua; MR, mitral regurgitation; ESRD, end-stage renal disease; SLE, systemic lupus erythematosus; SCLC, small cell lung cancer; AI, adrenal insufficiency; ES, Evan's syndrome; HIV human immunodeficiency virus; AIDS, acquired immunodeficiency syndrome; T-PLL, T-cell prolymphocytic leukemia; CVA, cerebrovascular accident; ALL, acute lymphatic leukemia; HCV, hepatitis C virus; LTtx, liver transplantation; MDS, myelodysplastic syndrome; KTtx, kidney transplantation; CML, chronic myeloid leukemia; NPC, nasopharyngeal carcinoma; hip fx, hip fracture.

HScore is marked yellow if the value is ≥150 and <169, as this indicates a probability of 25-50% of an HLH diagnosis according to the original HScore validation [3]. HLH-04 criteria is also marked yellow in cases where there are four fulfilled, as guidelines suggest that patients displaying enough HLH-stigmata to entertain this diagnosis should be treated as such while definitive diagnostics are performed [103, 104].

References

1. Henter J-I, Horne A, Aricó M, Egeler RM, Filipovich AH, Imashuku S, et al. HLH-2004: Diagnostic and therapeutic guidelines for hemophagocytic lymphohistiocytosis. Pediatric Blood & Cancer. 2007;48(2):124-31.
2. Jordan MB, Allen CE, Weitzman S, Filipovich AH, McClain KL. How I treat hemophagocytic lymphohistiocytosis. Blood. 2011;118(15):4041-52.
3. Fardet L, Galicier L, Lambotte O, Marzac C, Aumont C, Chahwan D, et al. Development and Validation of the HScore, a Score for the Diagnosis of Reactive Hemophagocytic Syndrome. Arthritis & Rheumatology. 2014;66(9):2613-20.
4. Brastianos PK, Swanson JW, Torbenson M, Sperati J, Karakousis PC. Tuberculosis-associated haemophagocytic syndrome. Lancet Infect Dis. 2006;6(7):447-54.
5. Trovik LH, Sandnes M, Blomberg B, Holmaas G, Ahmed AB, Tvedt THA, et al. Hemophagocytic lymphohistiocytosis and miliary tuberculosis in a previously healthy individual: a case report. J Med Case Rep. 2020;14(1):217.
6. Troncoso Mariño A, Campelo Sánchez E, Martínez López de Castro N, Inaraja Bobo MT. Haemophagocytic syndrome and paradoxical reaction to tuberculostatics after treatment with infliximab. Pharm World Sci. 2010;32(2):117-9.
7. Singha A, Mukherjee A, Dasgupta R, Das T. A Case of Hemophagocytic Syndrome due to Tuberculosis: Uncommon Manifestation of a Common Disease. Case Rep Med. 2014;2014:613845.
8. Tseng YC, Sun HY, Tsai JH, Hung PP, Wang JT. TB-IRIS presenting with haemophagocytic lymphohistiocytosis in a non-HIV-infected male. Int J Tuberc Lung Dis. 2017;21(10):1183-4.

9. Wong CK, Wong BC, Chan KC, Joynt GM, Yap FY, Lam CW, et al. Cytokine profile in fatal human immunodeficiency virus tuberculosis Epstein-Barr virus associated hemophagocytic syndrome. Arch Intern Med. 2007;167(17):1901-3.
10. Zhang Y, Liang G, Qin H, Li Y, Zeng X. Tuberculosis-associated hemophagocytic lymphohistiocytosis with initial presentation of fever of unknown origin in a general hospital: An analysis of 8 clinical cases. Medicine (Baltimore). 2017;96(16):e6575.
11. Asaji M, Tobino K, Murakami K, Goto Y, Sueyasu T, Nishizawa S, et al. Miliary Tuberculosis in a Young Woman with Hemophagocytic Syndrome: A Case Report and Literature Review. Intern Med. 2017;56(12):1591-6.
12. Balasubramanian S, Kaarthigeyan K, Aparna V, Srinivas S. Tuberculosis associated hemophagocytic syndrome in infancy. Indian Pediatr. 2008;45(7):593-5.
13. Deshpande A, Nayar PS, Pradhan AM, Manchanda RV. Miliary tuberculosis with hemophagocytosis in a two months old infant. Indian J Hematol Blood Transfus. 2010;26(3):115-7.
14. Seo JH, Lee JA, Kim DH, Cho J, Lim JS. Tuberculosis-associated hemophagocytic lymphohistiocytosis in adolescent diagnosed by polymerase chain reaction. Korean J Pediatr. 2016;59(1):43-6.
15. Okascharoen C, Nuntnarumit P, Sirinavin S. Neonatal tuberculosis associated with shock, disseminated intravascular coagulation, hemophagocytic syndrome, and hypercalcemia: a case report. J Perinatol. 2003;23(1):79-81.
16. Chen CH, Fang YH, Chiang PM, Lin DT, Huang LM. Disseminated tuberculosis presenting as multiple hepatosplenic microabscesses and pancytopenia in a teenage boy. J Formos Med Assoc. 2004;103(12):939-42.
17. Gupta AP, Parate SN, Bobhate SK. Hemophagocytic syndrome: a cause for fatal outcome in tuberculosis. Indian J Pathol Microbiol. 2009;52(2):260-2.
18. Dilber E, Erduran E, Kalyoncu M, Aynaci FM, Okten A, Ahmetoğlu A. Hemophagocytic syndrome as an initial presentation of miliary tuberculosis without pulmonary findings. Scand J Infect Dis. 2002;34(9):689-92.
19. Shaw PH, Brown D, Shulman ST. Tuberculosis-associated hemophagocytic syndrome in an infant. Pediatr Infect Dis J. 2000;19(5):475-7.
20. Balkis MM, Bazzi L, Taher A, Salem Z, Uthman I, Kanj N, et al. Severe hemophagocytic syndrome developing after treatment initiation for disseminated Mycobacterium tuberculosis: case report and literature review. Scand J Infect Dis. 2009;41(6-7):535-7.
21. Chen L, Weng H, Li H, Huang J, Pan J, Huang Y, et al. Potential killer in the ICU-severe tuberculosis combined with hemophagocytic syndrome: A case series and literature review. Medicine (Baltimore). 2017;96(49):e9142.
22. Erdoğan S, Çakır D, Bozkurt T, Karakayalı B, Kalın S, Koç B, et al. Hemophagocytic Lymphohistiocytosis Related to Tuberculosis Disease. Indian J Crit Care Med. 2020;24(1):63-5.
23. Geerdes-Fenge HF, Löbermann M, Hemmer CJ, Benedek O, Reisinger EC. Tuberculosis-associated hemophagocytic lymphohistiocytosis with subsequent unmasking cryptococcal immune reconstitution inflammatory syndrome (IRIS) in an HIV-negative man. Infection. 2019;47(1):129-33.
24. Haque WM, Shuvo ME, Rahim MA, Mitra P, Samad T, Haque JA. Haemophagocytic syndrome in an adult suffering from pyrexia of unknown origin: an uncommon presentation of tuberculosis: a case report. BMC Res Notes. 2017;10(1):110.
25. Shoureshi P, Ruiz J, Abdulzahir A, Bisch AL, Naddaf N, Gisel J. Tuberculosis-associated HLH in a patient with chronic kidney disease on haemodialysis. Oxf Med Case Reports. 2020;2020(10):omaa082.
26. Su NW, Chen CK, Chen GS, Hsieh RK, Chang MC. A case of tuberculosis-induced hemophagocytic lymphohistiocytosis in a patient under hemodialysis. Int J Hematol. 2009;89(3):298-301.

27. Shiu SJ, Li TT, Lee BJ, Fu PK, Wang CY, Shiu SI. Miliary Tuberculosis-Related Acute Respiratory Distress Syndrome Complicated with Hemophagocytic Lymphohistiocytosis Syndrome. Case Rep Infect Dis. 2019;2019:9501610.
28. Hashmi HRT, Mishra R, Niazi M, Venkatram S, Diaz-Fuentes G. An Unusual Triad of Hemophagocytic Syndrome, Lymphoma and Tuberculosis in a Non-HIV Patient. Am J Case Rep. 2017;18:739-45.
29. Dey A, Shah I, Paikrao P, Iyenger V. Tuberculosis with hemophagocytic lymphohistiocytosis in an infant. Indian J Pediatr. 2014;81(2):214-5.
30. Hauch H, Skrzypek S, Woessmann W, Lehmberg K, Ehl S, Speckmann C, et al. Tuberculosis-Associated HLH in an 8-Month-Old Infant: A Case Report and Review. Front Pediatr. 2020;8:556155.
31. Koulmane Laxminarayana SL, Nagaraju SP, Prabhu Attur R, Manohar C, Parthasarathy R, Chari B. Hemophagocytic lymphohistiocytosis: An unusual presentation of tuberculosis in hemodialysis patients. Hemodial Int. 2015;19(3):E16-9.
32. Maheshwari P, Chhabra R, Yadav P. Perinatal tuberculosis associated hemophagocytic lymphohistiocytosis. Indian J Pediatr. 2012;79(9):1228-9.
33. Martínez-Pillado M, Varela-Durán M, Said-Criado I, Díaz-Parada P, Rodríguez-Losada M, Mendoza-Pintos M. Disseminated tuberculosis and hemophagocytic syndrome although TB prophylaxis in patients with inflammatory bowel disease treated with Infliximab. IDCases. 2019;16:e00518.
34. Mbizvo GK, Lentell IC, Leen C, Roddie H, Derry CP, Duncan SE, et al. Epilepsia partialis continua complicated by disseminated tuberculosis and hemophagocytic lymphohistiocytosis: a case report. J Med Case Rep. 2019;13(1):191.
35. Ohata S, Hara K, Arai T, Takayoshi T, Nishiyama K, Yasutomo Y, et al. A case of pulmonary tuberculosis diagnosed in a patient with manifestations of haemophagocytic lymphohistiocytosis. Oxf Med Case Reports. 2019;2019(3):omz013.
36. Padhi S, Varghese RG, Ramdas A, Phansalkar MD, Sarangi R. Hemophagocytic lymphohistiocytosis: critical reappraisal of a potentially under-recognized condition. Front Med. 2013;7(4):492-8.
37. Parsi M, Dargan K. Hemophagocytic Lymphohistiocytosis Induced Cytokine Storm Secondary to Human Immunodeficiency Virus Associated Miliary Tuberculosis. Cureus. 2020;12(1):e6589.
38. Rathnayake PV, Kularathne WK, De Silva GC, Athauda BM, Nanayakkara SN, Siribaddana A, et al. Disseminated tuberculosis presenting as hemophagocytic lymphohistiocytosis in an immunocompetent adult patient: a case report. J Med Case Rep. 2015;9:294.
39. Seminari E, Contardi G, Rubert L, Fronti E, Comoli P, Minoli L, et al. Tuberculosis-induced haemophagocytic syndrome in a patient on haemodialysis treated with anti-thymocyte globulin. Int J Tuberc Lung Dis. 2014;18(2):248-9.
40. Au WY, Kwong YL, Yuen KY. Hemophagocytosis in the peripheral blood due to tuberculosis mycobacteremia. Am J Med. 2005;118(11):1298-9.
41. Campo E, Condom E, Miro MJ, Cid MC, Romagosa V. Tuberculosis-associated hemophagocytic syndrome. A systemic process. Cancer. 1986;58(12):2640-5.
42. Chien CC, Chiou TJ, Lee MY, Hsiao LT, Kwang WK. Tuberculosis-associated hemophagocytic syndrome in a hemodialysis patient with protracted fever. Int J Hematol. 2004;79(4):334-6.
43. Shin BC, Kim SW, Ha SW, Sohn JW, Lee JM, Kim NS. Hemophagocytic syndrome associated with bilateral adrenal gland tuberculosis. Korean J Intern Med. 2004;19(1):70-3.
44. Wang YH, Ba JH, Shi XW, Wu BQ. Successful treatment of mycobacterial infection associated hemophagocytic lymphohistiocytosis with etoposide and anti-tuberculous therapy: a case report. BMC Infect Dis. 2020;20(1):321.

45. Elhence A, Aggarwal A, Goel A, Aggarwal M, Das P, Shalimar. Granulomatous Tubercular Hepatitis Presenting as Secondary Hemophagocytic Lymphohistiocytosis: A Case Report and Systematic Review of the Literature. J Clin Exp Hepatol. 2021;11(1):149-53.
46. Shi YF, Shi XH, Zhang Y, Chen JX, Lai WX, Luo JM, et al. Disseminated Tuberculosis Associated Hemophagocytic Lymphohistiocytosis in a Pregnant Woman With Evans syndrome: A Case Report and Literature Review. Front Immunol. 2021;12:676132.
47. Mandal A, Jat KR, Singh A, Mridha AR, Kabra SK. Autoimmune haemolytic anaemia and haemophagocytic lymphohistiocytosis in an adolescent boy with tuberculosis: an unusual association. Trop Doct. 2017;47(3):249-53.
48. Eliopoulos G, Vaiopoulos G, Kittas C, Fessas P. Tuberculosis associated hemophagocytic syndrome complicated with severe bone marrow failure and disseminated intravascular coagulation. Nouv Rev Fr Hematol. 1992;34(3):273-6.
49. Koduri PR, Carandang G, DeMarais P, Patel AR. Hyperferritinemia in reactive hemophagocytic syndrome report of four adult cases. Am J Hematol. 1995;49(3):247-9.
50. Avasthi R, Mohanty D, Chaudhary SC, Mishra K. Disseminated tuberculosis: interesting hematological observations. J Assoc Physicians India. 2010;58:243-4.
51. Basu S, Mohan H, Malhotra H. Pancytopenia due to hemophagocytic syndrome as the presenting manifestation of tuberculosis. J Assoc Physicians India. 2000;48(8):845-6.
52. Browett PJ, Varcoe AR, Fraser AG, Ellis-Pegler RB. Disseminated tuberculosis complicated by the hemophagocytic syndrome. Aust N Z J Med. 1988;18(1):79-80.
53. Aggarwal P, Kumar G, Dev N, Kumari P. Haemophagocytic lymphohistiocytosis: a cause for rare but fatal outcome in tuberculosis. BMJ Case Rep. 2012;2012.
54. Barnes N, Bellamy D, Ireland R, Parsons V, Costello J. Pulmonary tuberculosis complicated by haemophagocytic syndrome and rifampicin-induced tubulointerstitial nephritis. Br J Dis Chest. 1984;78(4):395-403.
55. Bauer MP, van Burgel ND, Marijt WA, van Dissel JT, von dem Borne PA. Fever, shock, and pancytopenia in a patient treated with alemtuzumab. Clin Infect Dis. 2009;49(10):1540; 616-7.
56. Castellano I, Gómez-Martino JR, Hernández T, Mateos L, Argüello C. Hemophagocytic syndrome as an unusual form of presentation of tuberculosis in a hemodialysis patient: case report and review of the literature. Am J Nephrol. 2000;20(3):214-6.
57. Cassim KM, Gathiram V, Jogessar VB. Pancytopaenia associated with disseminated tuberculosis, reactive histiocytic haemophagocytic syndrome and tuberculous hypersplenism. Tuber Lung Dis. 1993;74(3):208-10.
58. Cherif E, Bel Feki N, Ben Hassine L, Khalfallah N. Haemophagocytic syndrome with disseminated intravascular coagulation associated with tuberculosis. BMJ Case Rep. 2013;2013.
59. Claessens YE, Pene F, Tulliez M, Cariou A, Chiche JD. Life-threatening hemophagocytic syndrome related to mycobacterium tuberculosis. Eur J Emerg Med. 2006;13(3):172-4.
60. Goto S, Aoike I, Shibasaki Y, Morita T, Miyazaki S, Shimizu T, et al. A successfully treated case of disseminated tuberculosis-associated hemophagocytic syndrome and multiple organ dysfunction syndrome. Am J Kidney Dis. 2001;38(4):E19.
61. Hui YM, Pillinger T, Luqmani A, Cooper N. Haemophagocytic lymphohistiocytosis associated with Mycobacterium tuberculosis infection. BMJ Case Rep. 2015;2015.
62. Kaur N, Britton PN, Isaacs D, Ging J, Campbell DE. Haemophagocytic lymphohistiocytosis secondary to presumed congenital tuberculosis in a neonate. J Paediatr Child Health. 2019;55(8):988-92.

63. Ko YC, Lee CT, Cheng YF, Hung KH, Kuo CY, Huang CC, et al. Hypercalcaemia and haemophagocytic syndrome: rare concurrent presentations of disseminated tuberculosis in a dialysis patient. Int J Clin Pract. 2004;58(7):723-5.
64. Lee SW, Wang CY, Lee BJ, Kuo CY, Kuo CL. Hemophagocytic syndrome in miliary tuberculosis presenting with noncaseating granulomas in bone marrow and liver. J Formos Med Assoc. 2008;107(6):495-9.
65. Long B, Cheng L, Lai SP, Zhang JW, Sun YL, Lai WX, et al. Tuberculosis-associated hemophagocytic lymphohistiocytosis in an umbilical cord blood transplant recipient. Clin Chim Acta. 2017;468:111-3.
66. Tesi B, Sieni E, Neves C, Romano F, Cetica V, Cordeiro AI, et al. Hemophagocytic lymphohistiocytosis in 2 patients with underlying IFN-γ receptor deficiency. J Allergy Clin Immunol. 2015;135(6):1638-41.
67. Verma T, Aggarwal S. Childhood tuberculosis presenting with haemophagocytic syndrome. Indian J Hematol Blood Transfus. 2012;28(3):178-80.
68. Mancebo E, Allende LM, Guzmán M, Paz-Artal E, Gil J, Urrea-Moreno R, et al. Familial hemophagocytic lymphohistiocytosis in an adult patient homozygous for A91V in the perforin gene, with tuberculosis infection. Haematologica. 2006;91(9):1257-60.
69. Naha K, Dasari S, Vivek G, Prabhu M. Disseminated tuberculosis presenting with secondary haemophagocytic lymphohistiocytosis and Poncet's disease in an immunocompetent individual. BMJ Case Rep. 2013;2013.
70. Osowicki J, Wang S, McKenzie C, Marshall C, Gard J, Ke Juin W, et al. Congenital Tuberculosis Complicated by Hemophagocytic Lymphohistiocytosis. Pediatr Infect Dis J. 2016;35(1):108-10.
71. Rodríguez-Medina B, Blanes M, Vinaixa C, Aguilera V, Rubín A, Prieto M, et al. Haemophagocytic syndrome in a liver transplant patient during treatment with Telaprevir. Ann Hepatol. 2013;12(6):974-8.
72. Ruiz-Argüelles GJ, Arizpe-Bravo D, Garcés-Eisele J, Sánchez-Sosa S, Ruiz-Argüelles A, Ponce-de-León S. Tuberculosis-associated fatal hemophagocytic syndrome in a patient with lymphoma treated with fludarabine. Leuk Lymphoma. 1998;28(5-6):599-602.
73. Sáez-González E, Salavert M, Cerrillo E, Moret I, Iborra M, Nos P, et al. Secondary Haemophagocytic Syndrome and Overlapping Immune Reconstitution Syndrome: Life-Threatening Complications of Anti-TNF-α Treatment for Crohn's Disease. Am J Gastroenterol. 2019;114(1):177-9.
74. Satomi A, Nagai S, Nagai T, Niikura K, Ideura T, Ogata H, et al. Effect of plasma exchange on refractory hemophagocytic syndrome complicated with myelodysplastic syndrome. Ther Apher. 1999;3(4):317-9.
75. Baraldès MA, Domingo P, González MJ, Aventin A, Coll P. Tuberculosis-associated hemophagocytic syndrome in patients with acquired immunodeficiency syndrome. Arch Intern Med. 1998;158(2):194-5.
76. Brito-Zerón P, Bosch X, Pérez-de-Lis M, Pérez-Álvarez R, Fraile G, Gheitasi H, et al. Infection is the major trigger of hemophagocytic syndrome in adult patients treated with biological therapies. Semin Arthritis Rheum. 2016;45(4):391-9.
77. Hansen S, Alduaij W, Biggs CM, Belga S, Luecke K, Merkeley H, et al. Ruxolitinib as adjunctive therapy for secondary hemophagocytic lymphohistiocytosis: A case series. Eur J Haematol. 2021;106(5):654-61.
78. Weintraub M, Siegman-Igra Y, Josiphov J, Rahmani R, Liron M. Histiocytic hemophagocytosis in miliary tuberculosis. Arch Intern Med. 1984;144(10):2055-6.
79. Yang CW, Lee JH, Kim YG, Kim YO, Lee SH, Kim BK, et al. Tuberculosis-associated hemophagocytic syndrome in a hemodialysis patient: case report and review of the literature. Nephron. 1996;72(4):690-2.
80. Quiquandon I, Plantier I, Hatron PY, Chassaing O, Bauters F, Desablens B, et al. Tuberculosis associated haemophagocytic syndrome: two cases with a favourable outcome. Nouv Rev Fr Hematol. 1995;37(2):149-52.

81. Rosales-Castillo A, López-Ruz M. Miliary tuberculosis complicated with acute respiratory distress syndrome and hemophagocytic lymphohistiocytosis syndrome in an immunocompetent patient. Med Clin (Barc). 2020;157(9):454-5.
82. Subhash HS, Sowmya S, Sitaram U, Cherian AM. Tuberculosis associated haemophagocytic syndrome. J Postgrad Med. 2001;47(3):220.
83. Jaiswal A, Mallya V, Singh V, Walia M, Khurana N. Hemophagocytic lymphohistiocytosis secondary to multiple infections: Case report of a rare entity. Indian J Pathol Microbiol. 2017;60(1):137-8.
84. Fernández AA, de Velasco Pérez DF, Fournier MC, Moreno Del Prado JC, Torras BP, Cañete Palomo ML. Hemophagocytic syndrome secondary to tuberculosis at 24-week gestation. Int J Mycobacteriol. 2017;6(1):108-10.
85. Undar L, Karpuzoğlu G, Karadoğan I, Gelen T, Artvinli M. Tuberculosis-associated haemophagocytic syndrome: a report of two cases and a review of the literature. Acta Haematol. 1996;96(2):73-8.
86. Lam KY, Ng WF, Chan AC. Miliary tuberculosis with splenic rupture: a fatal case with hemophagocytic syndrome and possible association with long standing sarcoidosis. Pathology. 1994;26(4):493-6.
87. Chan JK, Ng CS, Law CK, Ng WF, Wong KF. Reactive hemophagocytic syndrome: a study of 7 fatal cases. Pathology. 1987;19(1):43-50.
88. Shea YF, Chan JF, Kwok WC, Hwang YY, Chan TC, Ni MY, et al. Haemophagocytic lymphohistiocytosis: an uncommon clinical presentation of tuberculosis. Hong Kong Med J. 2012;18(6):517-25.
89. Padhi S, Ravichandran K, Sahoo J, Varghese RG, Basheer A. Hemophagocytic lymphohistiocytosis: An unusual complication in disseminated Mycobacterium tuberculosis. Lung India. 2015;32(6):593-601.
90. Ranjan A, Pal RS, Kumar A, Chandra Ojha U. Haemophagocytic lymphohistiocytosis (HLH) secondary to miliary tuberculosis. Indian J Tuberc. 2020;67(3):366-70.
91. Vaiphei K, Ahuja V, Sinha SK, Bhasin DK. Prolonged fever with lymph nodal and liver involvement in a chronic alcoholic man. Indian J Gastroenterol. 2008;27(3):123-9.
92. Talluri MR, Uppin S, Paritala V, Kumar N, Challa S, Rao N, et al. Miliary Tuberculosis: A Rare Cause of Hemophagocytic Lymphohistiocytosis. Chest. 2013;144(4, Supplement):211A.
93. Halabi H, Hafiz W, Bawayan M, Maulawi A, Almoallim H. Mycobacterium Tuberculosis-Associated Hemophagocytic Syndrome in Systemic Lupus Erythematosus: A Case Report/Sistemik Lupus Eritematözde Mycobacterium Tuberculosis ile Iliskili Hemofagositik Sendrom: Olgu Sunumu. Turkish journal of rheumatology. 2012;27(4):267.
94. Jain D, Dash S. Pancytopenia due to extensive hemophagocytosis following anti-tubercular treatment. Am J Hematol. 2004;75(2):118-9.
95. Khan F, Fawzy Z, Siddiqui I, Yassin M. Hemophagocytosis and miliary tuberculosis in a patient in the intensive care unit. Indian Journal of Critical Care Medicine. 2006;10(2):112-4.
96. Monier B, Fauroux B, Chevalier JY, Leverger G, Nathanson M, Costil J, et al. Miliary tuberculosis with acute respiratory failure and histiocytic hemophagocytosis. Successful treatment with extracorporeal lung support and epipodophyllotoxin VP 16-213. Acta Paediatr. 1992;81(9):725-7.
97. Karras A, Thervet E, Legendre C. Hemophagocytic syndrome in renal transplant recipients: report of 17 cases and review of literature. Transplantation. 2004;77(2):238-43.
98. Kürşat S, Cağirgan S, Ok E, Unsal A, Tokat Y, Saydam G, et al. Haemophagocytic-histiocytic syndrome in renal transplantation. Nephrology Dialysis Transplantation. 1997;12(5):1058-60.
99. Joshi R, Phatarpekar A, Currimbhoy Z, Desai M. Haemophagocytic lymphohistiocytosis: a case series from Mumbai. Ann Trop Paediatr. 2011;31(2):135-40.

100. McCall CM, Mudali S, Arceci RJ, Small D, Fuller S, Gocke CD, et al. Flow cytometric findings in hemophagocytic lymphohistiocytosis. Am J Clin Pathol. 2012;137(5):786-94.
101. Modi C, Dhamne A, Rege JD. Haemophagocytosis in tuberculosis--a case report. Indian J Pathol Microbiol. 2003;46(3):463-5.
102. Akinbami LJ, Selby DM, Slonim AD. Hepatosplenomegaly and pulmonary infiltrates in an infant. The Journal of Pediatrics. 2001;139(1):124-9.
103. Jordan MB, Allen CE, Greenberg J, Henry M, Hermiston ML, Kumar A, et al. Challenges in the diagnosis of hemophagocytic lymphohistiocytosis: Recommendations from the North American Consortium for Histiocytosis (NACHO). Pediatr Blood Cancer. 2019;66(11):e27929.
104. La Rosée P, Horne A, Hines M, von Bahr Greenwood T, Machowicz R, Berliner N, et al. Recommendations for the management of hemophagocytic lymphohistiocytosis in adults. Blood. 2019;133(23):2465-77.
